# Clinical utility of ultra-rapid whole-genome sequencing in an infant with atypical presentation of *WT1*-associated nephrotic syndrome type 4

**DOI:** 10.1101/mcs.a005470

**Published:** 2020-08

**Authors:** Erica Sanford, Terence Wong, Katarzyna A. Ellsworth, Elizabeth Ingulli, Stephen F. Kingsmore

**Affiliations:** 1 Rady Children's Institute of Genomic Medicine, Rady Children's Hospital–San Diego, San Diego, California 92123, USA;; 2 Division of Pediatric Intensive Care Medicine, Department of Pediatrics, University of California San Diego, La Jolla, California 92093, USA;; 3 Division of Pediatric Nephrology, Department of Pediatrics, University of California San Diego, La Jolla, California 92093, USA

**Keywords:** abnormal renal physiology, abnormality of urine homeostasis, anuria, chronic metabolic acidosis, episodic vomiting, global glomerulosclerosis, hypertensive crisis, hyponatremia, intermittent diarrhea, nephrotic range proteinuria

## Abstract

Relatively little is known about phenotypic variability in nonsyndromic nephropathy associated with the gene encoding the WT1 transcription factor. We report a 12-mo-old female who presented with vomiting, diarrhea, and fatigue in the setting of renal failure and malignant hypertension. Trio ultra-rapid whole-genome sequencing identified a novel, likely pathogenic, de novo missense variant (c.485T > A, p.Val162Asp) in *WT1* in 46 h, consistent with a diagnosis of nephrotic syndrome type 4 (NPHS4; OMIM 256370). This disorder typically presents with nephrotic syndrome (gross proteinuria, hypoalbuminemia, and edema). Rapid diagnosis had an immediate impact on her clinical management in the pediatric intensive care unit. Diagnostic renal biopsy was avoided, and placement of permanent dialysis access, a gastrostomy tube, and bilateral nephrectomy were accelerated. This report expands the presenting phenotype of nonsyndromic nephrotic syndrome and/or renal failure due to heterozygous variants in *WT1* (NPHS4). It also highlights the relationship between time to genomic diagnosis and clinical utility in critically ill infants.

## INTRODUCTION

Disease-causing variants in the zinc finger DNA-binding protein WT1 are associated with five related autosomal dominant disorders: type 1 Wilms’ tumor (OMIM 194070), Denys–Drash syndrome (OMIM 194080), Frasier syndrome (OMIM 136680), Meacham syndrome (OMIM 608978), and nephrotic syndrome type 4 (NPHS4; OMIM 256370). WT1 is essential for normal development and function of the urogenital tract and mediates mesenchymal–epithelial transition and differentiation during development of kidneys and gonads (Pritchard-Jones et al. 1990; Morrison et al. 2008; Kriedberg 2010; Lipska et al. 2014). In mature nephrons, WT1 is predominantly expressed in glomerular podocytes and is required for podocyte differentiation, structure, function, and maintenance (Pritchard-Jones et al. 1990; Niaudet and Gubler 2006; Benetti et al. 2010; Toska and Roberts 2014). WT1 is a transcription factor that contains an amino-terminal transactivation domain (corresponding to *WT1* exon 1) and a carboxy-terminal DNA-binding domain comprised of four zinc fingers (corresponding to *WT1* exons 7–10) (Lipska et al. 2014; Dome and Huff 2016). These domains permit WT1 to act as both a transcriptional activator and repressor (Morrison et al. 2008; Toska and Roberts 2014). Genes regulated by *WT1* encode transcription factors, growth factors, growth factor receptors, and podocyte proteins (Wagner et al. 2006; Morrison et al. 2008) involved in the function of the glomerular filtration barrier (Wagner et al. 2006; Morrison et al. 2008; Benetti et al. 2010).

Nephrotic syndrome (NS) refers to the combination of heavy proteinuria, hypoalbuminemia, and edema and results from malfunction of the glomerular filter (Downie et al. 2017). Genetic defects in *WT1* are associated both with isolated nephrotic syndrome (NPHS4) and syndromic NS with disorders of sexual development and nephroblastoma or gonadoblastoma (Denys–Drash syndrome, Frasier syndrome) and a wider array of developmental defects (Meacham syndrome). NPHS4, previously called sporadic isolated steroid-resistant NS isolated diffuse mesangial sclerosis, is associated with rapid progression to end-stage renal disease (ESRD) early in life (Jeanpierre et al. 1998; Schumacher et al. 1998; Ito et al. 2001, 2010; Benetti et al. 2010; Yang et al. 2013; Lipska et al. 2014; Dome and Huff 2016). The typical renal histopathology in NPHS4 is diffuse mesangial sclerosis, although focal segmental glomerulosclerosis has also been described.

Here we report a critically ill infant who was diagnosed by ultra-rapid whole-genome sequencing (urWGS) in 46 h with a novel *WT1* missense variant that was subsequently clinically interpreted to be a diagnosis of NPHS4 (Online Mendelian Inheritance in Man 2020). The novel clinical presentation in this child expands the spectrum of clinical phenotypes in NPHS4.

## RESULTS

### Clinical Presentation and Family History

The patient was a previously healthy 12-mo-old female who presented to the emergency room with 2 wk of vomiting, diarrhea, and fatigue (Table 1). Review of systems, birth history, development, and family history were unremarkable. Blood pressure was grossly elevated (161/71 mm Hg; 50th percentile for age-, height-, and sex-matched patients is 84/39). Her height was 70.5 cm (8th percentile) and weight was 6.78 kg (less than the 1st percentile). Her weight had been 7.17 kg (5th percentile) 3 wk earlier. Otherwise, physical examination was normal. Notably, the patient had normal female genitalia, did not have edema, and appeared mildly dehydrated. Laboratory studies were remarkable for severe hyponatremia (serum sodium 107 mmol/L, reference range 131–145 mmol/L), renal failure (serum creatinine 6.35 mg/dL, reference range 0.10–0.60 mg/dL, and serum blood urea nitrogen 108 mg/dL, reference range 4–18 mg/dL), acidosis (venous pH 7.089, reference range 7.31–7.4L, and serum bicarbonate <5 mmol/L, reference range 18–27 mmol/L), anemia (hemoglobin 8.6 g/dL, reference range 10.5–14.0), thrombocytopenia (platelets 159,000/µL at presentation and 90,000/µL 5 h later, reference range 140,000–440,000), elevated serum lactate dehydrogenase (LDH; 1225 µ/L, reference range 550–1000 µ/L), hypocalcemia (calcium 6.9 mg/dL, reference range 8.8–10.8 mg/dL, and ionized calcium 0.94 mmol/L, reference range 1.10–1.35 mmol/L). Of note, serum albumin was within the normal range. Urinalysis was notable for both microscopic hematuria (44 erythrocytes/µL, reference range 0–12/µL) and proteinuria (969 mg/dL, reference range <12 mg/dL). Chest radiogram was normal.

**Table 1.** Phenotypes previously associated with nephrotic syndrome type 4 (NPHS4) and observed herein

Phenotype (HPO ID)	Present/Absent/Novel
Nephrotic syndrome (HP:0000100)	Absent^a^
Proteinuria (HP:0012593)	Present
Renal failure (HP:0000083)	Present
Diffuse mesangial sclerosis (HP:0001967)	Present
Focal segmental glomerulosclerosis (HP:0000097)	Absent
Onset in early childhood (n.a.)	Present
Vomiting (HP:0002013)	Novel
Diarrhea (HP:0002014)	Novel
Fatigue (HP:0012378)	Novel
Malignant hypertension (HP:0000822)	Novel
Hyponatremia (HP:0002902)	Novel
Metabolic acidosis (HP:0001942)	Novel

^a^Urine protein to creatinine ratio was in the nephrotic range, but edema and hypoalbuminemia were absent.

At presentation, vomiting and fatigue were considered to reflect symptomatic, severe hyponatremia and were treated with a hypertonic saline infusion (3%, 35 mL) (Lee et al. 2014) with change in serum osmolality from 259 mmol/kg to 265 mmol/kg. Her central venous pressure and urine output were within normal limits during the first ∼9 h following admission to the Pediatric Intensive Care Unit (6–12 cm H_2_O and 1 mL/kg/h, respectively). The urine protein to creatinine ratio was 78.1 (reference range <0.2, nephrotic range >2). Predicated on the history of good health and normal growth and development until 2 wk prior to admission, the initial clinical diagnosis was acute kidney injury with a differential diagnosis that included hemolytic uremic syndrome (HUS) and acute glomerulonephritis. Hypertension was treated with continuous intravenous nicardipine. After ∼9 h in the Pediatric Intensive Care Unit she became abruptly anuric. She was intubated and mechanically ventilated, a temporary dialysis catheter was placed, and continuous renal replacement therapy was initiated. Subsequently, parathyroid hormone was found to be elevated (378.5 pg/mL, reference range 8.5–72.5 pg/mL), suggesting that renal failure was more long-standing.

### Genomic Analyses

Trio (patient and parents) ultra-rapid whole-genome sequencing (urWGS) identified the etiology of her renal failure in 46 h. Of 14,687 nucleotide variants with potentially deleterious impact on proteins, the top ranked by disease phenotype match was a novel, heterozygous, de novo, missense variant, c.485T > A (p.Val162Asp), in *WT1* exon 1 (Table 2; PS2). This variant was absent from the gnomAD population database and thus was presumed rare (PM2). Multiple lines of computational evidence supported a deleterious effect of this variant (PP3). Therefore, based on the overall evidence (PS2, PM2, PP3), it was classified as likely pathogenic according to American College of Medical Genetics and Genomics (ACMG) and Association for Molecular Pathology (AMP) standards and guidelines (Richards et al. 2015).

**Table 2.** WT1 variant identified in the patient

Gene	Chromosome (GRCh37, hg19)	HGVS DNA reference	HGVS protein reference	Variant type	Predicted effect	Genotype (zygosity)	Parent of origin
*WT1* (ENST00000332351)	11:32456407	c.485T > A (NM_024426.5, c.500T > A)	p.Val162Asp (NP_077744.4, p.Val167Asp)	Substitution	Missense	Heterozygous	De novo

### Treatment Outcomes

Among WT1-related disorders, NPHS4 was considered the etiology of the patient's renal failure. The microangiopathic hemolytic anemia observed at presentation (manifested by thrombocytopenia, anemia, and elevated LDH) was considered to be secondary to severe hypertension. The molecular diagnosis clarified the patient's prognosis and treatment. Based on the NPHS4 diagnosis, she was categorized as having irreversible ESRD that would not respond to a trial of therapy, necessitating eventual renal transplant. A permanent hemodialysis catheter was placed surgically 2 d after the diagnosis.

**Figure 1.**
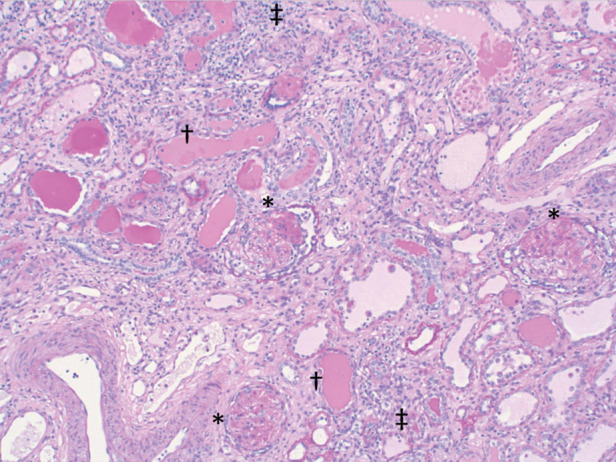
Photomicrograph of periodic acid–Schiff-stained kidney parenchyma showing extensive glomerulosclerosis (marked with *), consistent with diffuse mesangial sclerosis. Many renal tubules are dilated and show prominent thyroidization (marked with †) and atrophy. The interstitium shows marked chronic inflammation (marked with ‡).

Hypertension continued despite treatment with four antihypertensive drugs. In light of this and a risk of development of Wilms’ tumor, she received bilateral nephrectomy, peritoneal dialysis catheter placement, and gastrostomy tube 1 wk later. Kidney histology following nephrectomy revealed extensive bilateral global glomerulosclerosis consistent with NPHS4 (Fig. 1).

After 7 wk in hospital, the patient went home on peritoneal dialysis. Because her weight at discharge was 6.96 kg, peritoneal dialysis was planned until she reached the minimum weight for renal transplant (10 kg).

## DISCUSSION

Genetic diseases are common in infants and children in pediatric and neonatal intensive care units (PICUs and NICUs). Two recent studies suggested the incidence of genetic diseases among infants in regional NICUs to be ∼15% (Ceyhan-Birsoy et al. 2019; Kingsmore et al. 2019). The indications for rapid, diagnostic genomic sequencing in this population are broad (Supplemental Material). Succinctly, children who benefit from urWGS have diseases of unknown etiology in which the differential diagnosis is broad. The current case illustrates this nicely. The differential diagnosis in infantile renal failure is extremely broad. At presentation, it was unclear whether renal failure was acute or chronic, or whether it was of prerenal, renal, or postrenal origin. The differential diagnosis herein included congenital (infant-onset) and idiopathic (later childhood–onset) nephrotic syndrome, toxin-related kidney injury, hemolytic uremic syndrome (HUS), acute glomerulonephritis, interstitial nephritis, renal arterial or venous thrombosis, cortical necrosis, tubular necrosis, and congenital anomalies of kidney and urinary tract. Genetic disorders can underpin most of these. OMIM lists 383 genetic diseases associated with renal failure and 154 associated with nephrotic syndrome. Although imaging and laboratory studies can relatively rapidly rule out many pre- and post- and some intrarenal causes of renal failure, genome-wide sequencing is faster and less expensive than cascade testing for genetic causes (Stark et al. 2017).

urWGS is preferable to rapid genomic sequencing in children who are clinically unstable or with rapidly progressive conditions (Clark et al. 2019; Kingsmore et al. 2019). The current case met both criteria. She required mechanical ventilation for acute respiratory failure and hemodialysis for renal failure. urWGS returned a molecular diagnosis in 46 h. In contrast, rapid whole-genome sequencing or rapid whole-exome sequencing typically return results in 2 wk. In the absence of urWGS, it is likely that she would have undergone interim diagnostic renal biopsy, which carried increased risk of bleeding given severe hypertension, renal failure, and thrombocytopenia in a patient receiving continuous renal replacement therapy with regional anticoagulation. Avoidance of invasive biopsies has been observed recurrently in children receiving etiologic diagnoses for organ failure from urWGS (Farnaes et al. 2018).

Clinical diagnosis of genetic diseases is made considerably more difficult by atypical presentations. Infants, in particular, often present without the classic features of their illness (Clark et al. 2019). NPHS4, the diagnosis herein, typically presents with infant-onset nephrotic syndrome that rapidly progresses to renal failure. However, our patient did not exhibit hypoalbuminemia or edema, two of the three cardinal features of nephrotic syndrome. Instead she presented with vomiting, diarrhea, fatigue, and malignant hypertension. Atypical presentations decrease the posterior probability of the correct diagnosis within the differential diagnosis. To diagnose genetic disorders in infants with phenotype expansion requires a combination of hypothesis-free genome-wide sequencing, deep phenotyping, and nonpenalizing phenotype-matching algorithms (Clark et al. 2019).

The current case also illustrates how trio urWGS can enable diagnosis faster than singleton analysis. De novo variants, as herein, account for more than one-half of molecular diagnoses of genetic diseases in the United States (Willig et al. 2015; Farnaes et al. 2018; Kingsmore et al. 2019). De novo occurrence, with confirmed maternity and paternity, can promote missense variants of uncertain significance to likely pathogenicity under ACMG and AMP guidelines. In suspected de novo or compound heterozygous variants, singleton genomic sequencing must be supplemented by parental genotyping for confirmation of inheritance prior to reporting. Typically, this delays results by a week. Thus, in general, urWGS should be performed as trios.

Herein, the molecular diagnosis changed the patient's prognosis and treatment. Renal function can be improved by appropriate treatment in a few of the disorders on the original differential diagnosis herein (Dufek et al. 2018). In HUS and acute glomerulonephritis, for example, renal failure may respond to plasmapheresis or high-dose corticosteroids, respectively. In contrast, NPHS4 is associated with end-stage renal disease that does not respond to such therapies (Jeanpierre et al. 1998; Schumacher et al. 1998; Ito et al. 2001, 2010; Royer-Pokora et al. 2004; Hinkes et al. 2007; Chernin et al. 2010; Downie et al. 2017). Without urWGS, definitive diagnosis would have been delayed, and the patient may have received these therapies empirically without benefit and with significant potential iatrogenic effects. Thus, molecular diagnosis of NPHS4 potentially avoided futile, iatrogenic treatment. In particular, plasmapheresis is associated with ischemic stroke and high-dose corticosteroids with infections and impaired wound healing. Etiologic diagnosis was useful herein for prognostic clarification. Diagnosis of NPHS4 indicated that the patient would require long-term dialysis and renal transplant. This prognosis accelerated the placement of permanent dialysis access and a gastrostomy tube. Had a definitive diagnosis not been established, dialysis would probably have been performed expectantly with temporary access for a longer period of time with increased risk of complications. Molecular diagnosis also assisted in the decision to perform immediate nephrectomy. The primary indication for bilateral nephrectomy herein was uncontrolled, persistent hypertension. However, the perceived risk of nephroblastoma (Wilms’ tumor) later in life was a secondary indication, especially in the setting of immunosuppression and renal transplantation. Wilms’ tumor is associated with null variants in *WT1.* However, it is not unequivocally known whether missense variants in *WT1* confer increased risk of nephroblastoma (Ito et al. 2001).

## METHODS

Parental consent was obtained for genome sequencing. Trio urWGS was performed at Rady Children's Institute for Genomic Medicine as described (Kingsmore et al. 2019). Following DNA isolation from whole blood, sequencing libraries were generated using the TruSeq DNA PCR-Free Library Prep kit (Illumina) according to the manufacturer's instructions. Paired-end sequencing was performed on a NovaSeq 6000 instrument and S1 flow cell (Illumina; Table 3). Read alignment to the reference human genome assembly GRCh37 and single-nucleotide variant (SNV)/insertion-deletion (indel) calling was performed using the DRAGEN Bio-IT Platform (Illumina). Copy-number variant (CNV) calling was performed using CNVnator and Manta (Kingsmore et al. 2019). SNVs/indels and CNVs were annotated and analyzed using Fabric Enterprise version 6.5.2 (Fabric Genomics).

**Table 3.** Average whole-genome sequencing coverage

Individual	Whole-genome sequencing coverage
Proband	46.8-fold
Mother	42.9-fold
Father	42.0-fold

Human Phenotype Ontology (HPO) terms used during variant interpretation included renal insufficiency (HP:0000083), hypertension (HP:0000822), vomiting (HP:0002013), diarrhea (HP:0002014), hemolytic–uremic syndrome (HP:0005575), fatigue (HP:0012378), and abnormal circulating metabolite concentration (HP:0032180). SNVs/indels were filtered to retain variants with allelic balance between 0.3 and 0.7 and allele frequency <0.5% in Genome Aggregation Database (gnomAD), prioritized by Phevor Gene Rank (Kingsmore et al. 2019), and classified according to ACMG and AMP standards and guidelines (Richards et al. 2015). CNVs were filtered to retain variants within coding exons or near genes shown to have an established gene–disease association and classified according to ACMG standards and guidelines (Kearney et al. 2011). Variants of interest were orthogonally confirmed by Sanger sequencing.

## ADDITIONAL INFORMATION

### Data Deposition and Access

The causative variant has been submitted to ClinVar (https://www.ncbi.nlm.nih.gov/clinvar/) with accession number SCV007346664. Sequence data is not publicly available because sequencing was performed as a clinical test.

### Ethics Statement

Whole-genome sequencing was performed as a clinical diagnostic test. Research consent was not obtained.

### Acknowledgments

We thank Dr. Helen Harvey who provided care for this patient in the PICU and Dr. Jun Mo who provided the photomicrograph.

### Author Contributions

E.S., T.W., and S.F.K. wrote the manuscript. All authors reviewed and edited the manuscript.

### Funding

Funding for this study was provided by Rady Children's Institute for Genomic Medicine.

### Competing Interest Statement

The authors have declared no competing interest.

### Referees

Andrew Mallett

Anonymous

## Supplementary Material

Supplemental Material
