# Supplementary material for: Clinical utility of ultra-rapid whole-genome sequencing in an infant with atypical presentation of WT1-associated nephrotic syndrome type 4
Source: Cold Spring Harb Mol Case Stud. 2020 Aug;6(4):a005470. doi: 10.1101/mcs.a005470 (PMC7476414; doi:10.1101/mcs.a005470)
Supplement: Supplemental Material [file supp_6_4_a005470__index.html]

Supplemental Material 

# Clinical utility of ultra-rapid whole-genome sequencing in an infant with atypical presentation of *WT1*-associated nephrotic syndrome type 4

## Supplemental Material

- Supplemental\_Material.docx
