# Supplementary material for: Clinical utility of ultra-rapid whole-genome sequencing in an infant with atypical presentation of WT1-associated nephrotic syndrome type 4
Source: Cold Spring Harb Mol Case Stud. 2020 Aug;6(4):a005470. doi: 10.1101/mcs.a005470 (PMC7476414; doi:10.1101/mcs.a005470)
Supplement: Supplemental Material [file supp_mcs.a005470_Supplemental_Material.docx]

Supplemental Material: Indications for Rapid Whole Genome Sequencing (rWGS) for Diagnosis of Genetic Disorders

From: <https://www.blueshieldca.com/bsca/bsc/public/common/PortalComponents/provider/StreamDocumentServlet?fileName=PRV_WholeExome_Sequen.pdf>

rWGS, with trio testing when possible, may be considered medically necessary for the evaluation of critically ill infants or children less than 18 years of age in neonatal or pediatric intensive care with illness of unknown etiology when both of the following criteria are met:

• At least one of the following criteria is met:

- Multiple congenital anomalies
  - Specific malformations highly suggestive of a genetic etiology, including but not
    limited to any of the following:
    - Choanal atresia
    - Coloboma
    - Hirschsprung disease
    - Meconium ileus
  - An abnormal laboratory test suggests a genetic disease or complex metabolic
    phenotype, including but not limited to any of the following:
    - Abnormal newborn screen
    - Conjugated hyperbilirubinemia not due to total parental nutrition
      cholestasis
    - Hyperammonemia
    - Lactic acidosis not due to poor perfusion
    - Refractory or severe hypoglycemia
  - An abnormal response to standard therapy for a major underlying condition
  - Significant hypotonia
  - Persistent seizures
  - Infant with high risk stratification on evaluation for a Brief Resolved Unexplained Event
    (BRUE) with any of the following features:
    - Recurrent events without respiratory infection
    - Recurrent witnessed seizure like events
    - Required Cardiopulmonary Resuscitation
    - Significantly abnormal chemistry including but not limited to electrolytes,
      bicarbonate or lactic acid, venous blood gas, glucose, or other tests that suggest
      an inborn error of metabolism
  - Significantly abnormal electrocardiogram, including but not limited to possible channelopathies, arrhythmias, cardiomyopathies, myocarditis or structural heart disease
  - Family history of:
    - Arrhythmia
    - BRUE in sibling
    - Developmental delay
    - Inborn error of metabolism or genetic disease
    - Long QT syndrome
    - Sudden unexplained death (including unexplained car accident or
      drowning) in first- or second-degree family members before age 35, and
      particularly as an infant
- None of the following criteria apply regarding the reason for admission:
  - An infection with normal response to therapy
  - Confirmed genetic diagnosis explains illness
  - Hypoxic Ischemic Encephalopathy with a clear precipitating event
  - Isolated prematurity
  - Isolated Transient Tachypnea of the Newborn
  - Isolated unconjugated hyperbilirubinemia
  - Nonviable neonates

Organ Transplantation
Rapid WGS may be considered for approval in some cases prior to undergoing organ transplantation when documentation supports the urgent need for testing.

Separate CMA testing is considered not medically necessary with rWGS analysis. Note: rWGS analysis has the ability to detect most CNVs.

Rapid Whole Genome Sequencing
Rapid means an average turnaround time of less than 14 days, but usually less than 7 days.
Rapid results should be called to the clinician immediately if changes in management are likely.

For rapid WGS, the patient should be critically ill and in the Neonatal or Pediatric Intensive Care Unit when the test is ordered, but may be discharged before the results are delivered.
